# Cytotoxicity of Thiopurine Drugs in Patients with Inflammatory Bowel Disease

**DOI:** 10.3390/toxics10040151

**Published:** 2022-03-22

**Authors:** Oliwia Zakerska-Banaszak, Liliana Łykowska-Szuber, Michał Walczak, Joanna Żuraszek, Aleksandra Zielińska, Marzena Skrzypczak-Zielińska

**Affiliations:** 1 Institute of Human Genetics, Polish Academy of Sciences, Strzeszynska 32, 60-479 Poznan, Poland; o.zakerska.banaszak@gmail.com (O.Z.-B.); michal.walczak@igcz.poznan.pl (M.W.); joanna.zuraszek@igcz.poznan.pl (J.Ż.); aleksandra.zielinska@igcz.poznan.pl (A.Z.); 2 Department of Gastroenterology, Dietetics and Internal Diseases, Poznan University of Medical Sciences, Przybyszewskiego 49, 60-355 Poznan, Poland; lszuber@wp.pl

**Keywords:** thiopurines, azathioprine, mercaptopurine, thioguanine, cytotoxicity, inflammatory bowel disease, Crohn’s disease, ulcerative colitis, pharmacogenetics, therapeutic drug monitoring

## Abstract

The effectiveness of thiopurine drugs in inflammatory bowel disease (IBD) was confirmed more than a half-century ago. It was proven that these can be essential immunomodulatory medications. Since then, they have been used routinely to maintain remission of Crohn’s disease (CD) and ulcerative colitis (UC). The cytotoxic properties of thiopurines and the numerous adverse effects of the treatment are controversial. However, the research subject of their pharmacology, therapy monitoring, and the search for predictive markers are still very relevant. In this article, we provide an overview of the current knowledge and findings in the field of thiopurines in IBD, focusing on the aspect of their cytotoxicity. Due to thiopurines’ benefits in IBD therapy, it is expected that they will still constitute an essential part of the CD and UC treatment algorithm. More studies are still required on the modulation of the action of thiopurines in combination therapy and their interaction with the gut microbiota.

## 1. Introduction

As the principal representatives of thiopurines, 6-mercaptopurine (6MP) and its prodrug azathioprine (AZA) are primary immunomodulating agents. They are used for example to manage inflammatory bowel diseases (IBD), including ulcerative colitis (UC) and Crohn’s disease (CD), chronic inflammatory disorders of the gastrointestinal tract. Thiopurines were used to treat CD in the late 1960s and they are currently applied in around 60% of IBD patients [1,2]. Considering that patients in this group often require treatment throughout their life and the possibilities of pharmacotherapy alternatives are limited to specific groups of drugs—aminosalicylates, glucocorticosteroids, immunosuppressants, biological medicines and antibiotics—the position of thiopurines is well established, especially as second-line drugs in maintaining disease remission [3,4]. Although most patients with IBD respond well to treatment with thiopurines, nearly one-third of them have their medication modified or discontinued due to numerous adverse effects [5,6]. Thiopurine therapy certainly generates challenges for personalized medicine and raises many questions. When does the cytotoxic effect of those drugs appear in IBD patients and which solution can overcome adverse effects? How should the impact of thiopurine treatment be monitored? What tools can predict the response to this therapy? Our article aims to answer the above questions and present an up-to-date overview of the cytotoxicity of thiopurines in IBD patients.

## 2. Metabolism of Thiopurine Drugs

**Figure 1:**
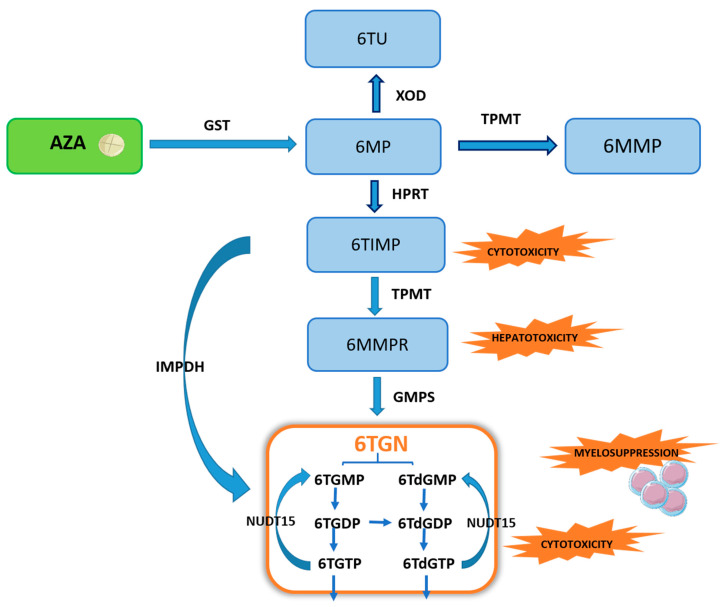
Scheme of the thiopurine biotransformation pathway. Explanation of abbreviations: AZA—azathioprine; GST—glutathione S-transferase; 6MP—6-mercaptopurine; XOD—xanthine oxidase/dehydrogenase; 6TUA—6-thiouric acid; TPMT—thiopurine methyltransferase; 6MMP—6-methylmercaptopurine; HPRT—hypoxanthine phosphoribosyltransferase; 6TIMP—6-thioinosine monophosphate; IMPDH—inosine monophosphate dehydrogenase; 6MMPR—6-methylmercaptopurine ribonucleotide; GMPS—guanosine monophosphate synthetase; 6TGN—6-thioguanine nucleotides; 6TGMP—6-thioguanosine monophosphate; 6TGDP—6-thio-guanosine diphosphate; 6TdGMP—6-thio-deoxyguanosine monophosphate; 6TdGDP—6-thiodeoxyguanosine diphosphate; 6TdGTP—6-thio-deoxyguanosine triphosphate; 6TGTP—6-thioguanosine triphosphate; NUDT15—nudix hydrolase motif 15.

Thiopurines undergo a complex metabolism, resulting in the formation of 6-thioguanine nucleotides (6TGN), the primary active therapeutic metabolite. Both AZA and 6MP, as prodrugs, undergo intracellular activation through a complex process, including the following enzymes: thiopurine methyltransferase (TPMT), xanthine oxidase/dehydrogenase (XOD), and hypoxanthine phosphoribosyltransferase (HPRT), forming three competitive pathways (Figure 1). In the first step, AZA is converted in the liver into 6MP, although only partly, by the enzyme glutathione S-transferase (GST), and evidence shows that it is also a non-enzymatic process [7,8]. Nevertheless, it is known that reduced activity of GST caused by mutations leads to reduced sensitivity to AZA in patients due to a lower concentration of active metabolites [9]. On the other hand, high activity of GST correlates with an increased risk of adverse effects and leukopenia during treatment with thiopurine drugs [10]. 

TPMT is responsible for the formation of inactive metabolites, 6-methylmercaptopurine (6MMP) and 6-methylmercaptopurine ribonucleotide (6MMPR), further converted to 6TGN by guanosine monophosphate synthetase (GMPS). However, it is worth noting that over 80% of the cases of thiopurine-induced hepatotoxicity are due to a high concentration of 6-MMPR in one week after treatment initiation. In addition, elevated 6-MMPR levels can contribute to the explanation of gastrointestinal intolerance and general malaise, the most common limiting adverse events of thiopurines [11]. There is evidence that hepatotoxicity also correlates with elevated 6-MMP levels [12]. TPMT constitutes a crucial enzyme regulating the biotransformation of thiopurines. The genetic polymorphism influencing TPMT enzyme activity may result in a clinical response and possible myelotoxicity among IBD patients [13]. High enzymatic activity of TPMT leads to a low therapeutic 6TGN level [14,15]. At the same time, patients with low activity of TPMT are susceptible to the myelotoxicity of thiopurine therapy.

HPRT enzyme catalyzes the conversion of 6MP to 6-thioinosine monophosphate (6TIMP), which is then metabolized directly into active 6TGN using an inosine monophosphate dehydrogenase (IMPDH) or TPMT and GMPS pathway [16,17]. XOD, known as the cytoplasmic enzyme, is involved in the liver and intestinal degradation of endogenous and exogenous substrates such as thiopurine. XOD metabolizes an integral part of 6MP into inactive 6-thiouric acid (6TUA). The activity of XOD is also regulated by different single nucleotide polymorphisms located in the gene coding for this enzyme and may explain inter-individual variations in enzyme activity. Poor XOD metabolizers have an increased risk of thiopurine adverse effects, whereas rapid metabolizers are the group with an increased risk of thiopurine therapy failure [18,19]. A recent significant discovery regarding the metabolic pathway of thiopurine drugs is the enzyme NUDT15, which catalyzes the hydrolysis of 6-thioguanosine triphosphate (6TGTP) to 6-thioguanosine monophosphate (6TGMP) [20]. A recent study in 54 Japanese IBD patients (27 UC, 27 CD) showed that a NUDT15 mutation leading to increased deoksythioguanosine (dTG) of DNA-incorporated may be responsible for thiopurine-induced leukopenia through cell apoptosis [21].

## 3. Cytotoxic Properties and the Mechanism of Action of Thiopurines

Like immunosuppressants, the general action of thiopurines is based on the inactivation of the critical T-cell processes that lead to inflammation. However, the exact mechanism and effect of thiopurines is not yet fully understood, and their cytotoxicity is determined by a combination of different factors.

### 3.1. Induction of Cell Apoptosis

One of the primary therapeutic metabolites, 6TGN (more specifically 6-thioguanine triphosphate, 6TGTP), binds to Ras-related C3 botulinum toxin substrate 1 (Rac1) as a competitive antagonist, an intracellular enzyme, small GTPase, involved in the activation of the inflammatory cascade (including NF-κB and STAT-3 pathways), and finally stimulates apoptosis of gut T cells [22]. Essentially, AZA converts a costimulatory signal into an apoptotic signal through this binding process. However, the affinity of 6TGTP to Rac1 is lower than that of its standard binding partner GTP. This can explain the delayed onset of the clinical activity of AZA [23]. 

Furthermore, it is a known mechanism of apoptosis induction by thiopurines via a mitochondrial pathway. Incorporating TG into mitochondrial DNA (mtDNA) causes accumulation of oxidized TG, which inhibits transcription and translation, and finally results in loss of mitochondrial function. It is speculated that this mechanism influences toxicity and myopathy induced by thiopurines [24]. 

### 3.2. Inhibition of DNA Replication and RNA Transcription 

There is evidence that deoxythioguanosine triphosphate (TdGTP), formed by ribonucleotide reductase from active metabolite 6TGTP during the biotransformation pathway, may be incorporated into DNA. TdGTP is a suitable substrate for DNA polymerases. Studies have shown that the incorporation frequency into the leukocyte DNA of patients undergoing treatment with MP varies between 1:32000 and 1:4000 thioguanine (TG) bases per guanine base [25,26]. In contrast, methylation of TG-thymine base pairs causes errors in the process of replication and transcription, also disrupting the DNA mismatch repair system, which results in DNA strand breaks, thus contributing to the thiopurine-mediated cytotoxicity effect. The studies also revealed that reactive oxygen generation and cell death are a consequence of TG incorporation into DNA [27,28].

During the incorporation of appropriate nucleotides into DNA, a particular role is played by the enzyme nudix hydrolase (nucleoside diphosphate-linked moiety X)-type motif 15 (NUDT15), which removes the wrong nucleotides from a cellular pole. NUDT15 can hydrolyze both TGTP and TdGTP (Figure 1). In vitro research has shown that NUDT15 knockdown results in significantly higher TGTP, TdGTP, and TG levels in DNA relative to controls [20,29]. Therefore, its role in the acute hematopoietic toxicity caused by thiopurine therapy has been proven.

### 3.3. Inhibition of De Novo Purine Synthesis

Thiopurines interfere with the synthesis of nucleic acids in dividing cells, acting mainly in the S phase. Mercaptopurine competes with hypoxanthine and guanine for hypoxanthine-guanyl phosphoribosyltransferase and is converted to thioinosine monophosphate (TIMP). This intracellular metabolite of mercaptopurine inhibits many reactions related to the metabolism of inosinic acid. S-methylation of TIMP with the participation of thiopurine S-methyltransferase (TPMT) leads to methylthioinosine monophosphate (MTIMP) formation. Both TIMP and MTIMP inhibit glutamine-5-phosphoribosyl pyrophosphate amidotransferase, the first enzyme in de novo synthesis of purine ribonucleotides [30].

Thiopurine metabolites vary in cytotoxicity, with methylthioinosine-mono-phosphate and thioguanosine-tri-phosphate being the most toxic and the methyl-thioguanosine nucleotides the least. Recent in vitro studies revealed that the most significant impact on cytotoxic properties of this group of drugs come from MTIMP and TG incorporation into DNA together as combined factors disturbing GTP signaling pathways [31].

## 4. Pharmacological Aspects and Clinical Characteristics of IBD Patients Treated with Thiopurines

Thiopurines are used as immunosuppressive drugs in both CD and UC. Guidelines for their application are set out in the European Crohn’s and Colitis Organisation (ECCO) Consensus [3,4].

### 4.1. Thiopurines in Crohn’s Disease (CD) Treatment

Experts suggest that thiopurines should not be used alone to induce remission in CD. Based on the available studies, the benefit of AZA monotherapy is not greater than that of a placebo [32]. However, the combination of thiopurines with infliximab (IFX) is recommended to induce remission in moderate to severe CD patients who have had an inadequate response to a conventional therapy and have not used AZA to date. Research by members of the Study of Biologic and Immunomodulator Naive Patients in Crohn’s Disease (SONIC), which compared the efficacy of IFX monotherapy with IFX in combination with AZA in patients who failed to respond to glucocorticoid therapy, showed that combination therapy had higher rates of clinical remission and mucosal remission at week 26 compared with IFX monotherapy (RR: 1.64; 95% CI: 1.07–2.53) (RR: 1.82; 95% CI: 1.01–3.26) [33]. However, in clinical practice there are cases where the patient uses thiopurines more often and the expected effect of maintaining remission is not achieved, which is recognized as an insufficient response to thiopurines. There are no reports that the combined therapy of thiopurines with IFX would be of benefit in achieving clinical remission in this group of patients, but doing so may be helpful in reducing IFX immunity. Naturally, we cannot forget about the immunogenicity of biological drugs. Therefore, in this aspect, the combined therapy should be considered by doctors individually for each patient [34].

Thiopurines have an undeniable and essential place in CD and are used to maintain remission in patients with a steroid-dependent form of the disease. Four hundred and eighty-nine patients were analyzed in the large meta-analysis of six studies. The advantage of AZA was demonstrated over the placebo in this group of patients (RR: 1.19; 95% CI: 1.05–1.34) [35]. However, it is not recommended to include thiopurines in all patients with newly diagnosed CD to maintain remission. The literature suggests that an early introduction of these drugs may contribute to the favorable course of the disease. However, conducted research has failed to confirm this thesis. In a study described by Panés et al., patients with a recent diagnosis of CD (<8 weeks) were randomized to two groups, one receiving a placebo and the other treated with AZA. At 76 weeks of therapy, no statistically significant differences were observed in the maintenance of disease remission between the two groups. The frequency of relapses and the need for glucocorticosteroids in both groups were comparable. Severe adverse effects occurred in 14 patients (20.6%) in the AZA group and 7 (11.1%) in the placebo group [36]. Its continuation is recommended in patients treated with thiopurines in long-term remission during thiopurine maintenance therapy. It is believed that there is a greater risk of the disease returning when treatment is discontinued [37].

### 4.2. Thiopurines in Ulcerative Colitis (UC) Treatment

In patients with steroid-dependent UC, thiopurine or IFX combined with thiopurine is also recommended. A study conducted by Ardizzone et al. showed that patients receiving prednisone at a dose of 40 mg/day and AZA at a quantity of 2 mg kg/day achieved significantly more steroid-free remission compared to the group receiving prednisone and 5-ASA 3.2 g per day (53% vs. 21%) [38]. Similarly, later research demonstrated an increase in remission without corticosteroids in UC patients treated with combination therapy after 16 weeks (39.7% vs. 22.1%, *p* = 0.017) [39]. Patients with UC display the same resistance to thiopurines as CD individuals. In such cases, it is recommended to use biological therapy and, if IFX is used, it should be in combination with a thiopurine. Another indication for using thiopurines in mild to moderate UC would be patients who experience frequent relapses using mesalazine or steroids. Retrospective studies indicated that the remission rate in patients treated with AZA was 58% and increased to 87% after six months of dosing [40]. In patients with severe relapsing disease responding to steroids, cyclosporine, IFX with thiopurine should be considered for use in maintaining remission. In a retrospection analysis of 622 patients with CD and UC, the remission rate after six months of AZA use was 64% and 87%, respectively. After discontinuation of AZA, the proportion of patients remaining in remission after 1, 3, and 5 years was 0.63, 0.44, and 0.35, respectively. The duration of AZA treatment did not affect the relapse rate after treatment was discontinued [40].

### 4.3. Combination Therapy of Thiopurines and Infliximab in IBD

Clinical practice and research show that the combination of IFX and thiopurines is more effective in inducing remission in CD and UC than monotherapy with both agents. Data on other varieties of other biologics and thiopurines are either missing or contradictory [41]. The results of combined IFX-thiopurines therapy can be explained by the best-documented theory of reducing the risk of immunogenicity, which decreases the production of anti-IFX antibodies. This effect is related to greater availability of the drug and thus a far better response to therapy [33]. Anti-IFX formation was observed as early as 18 days after the initiation of treatment. Therefore, to limit their use, thiopurines should be administered as soon as possible [42].

### 4.4. Safety and Adverse Effects of Thiopurine Treatment

**Figure 2:**
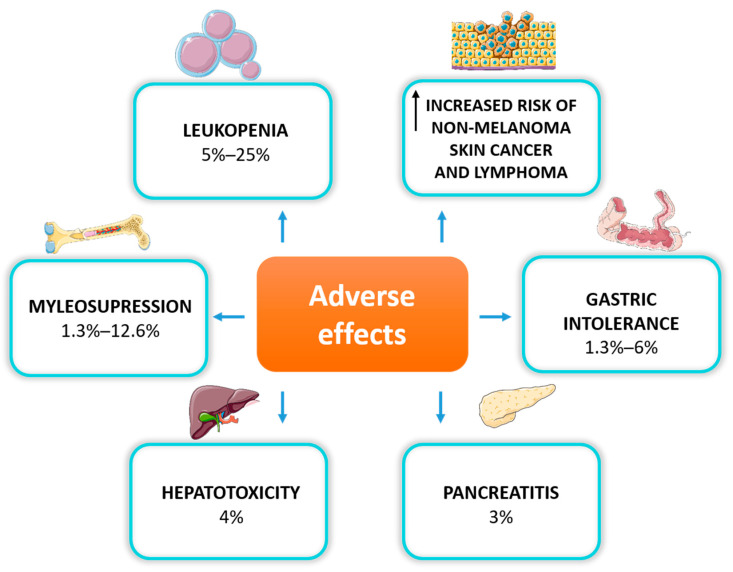
Adverse effects of thiopurine therapy in IBD patients.

When deciding to use AZA for IBD treatment, we should always consider its long-term safety. Data from observational population studies suggest caution and regular monitoring, especially for the risk of skin non-melanoma neoplasms and lymphomas in patients exposed to long-term treatment with thiopurines [43]. Adverse effects were reported in 9.0% (22/245) of patients treated with thiopurines vs. 2.9% (9/311) in those treated with a placebo [3]. Inappropriate therapeutic levels of 6TGN may trigger toxicity at distinct levels, including hepatotoxicity, myleosupression, pancreatitis, or gastrointestinal intolerance [5] (Figure 2).

The most commonly observed dose-dependent adverse reaction is myelosuppression, primarily manifested as leukopenia, which occurs in up to 20% of IBD patients due to *TPMT* gene polymorphism [44]. Thiopurines are also associated with myelosuppression, regardless of TPMT activity. Research has demonstrated that it can occur even many months after the initiation of the therapy, also in patients with normal TPMT phenotype [45]. Myelosuppression is the most severe hematological adverse drug reaction, leading to discontinuation of the treatment [46]. Thiopurines can also induce liver dysfunction by methylated intermediate metabolites, manifested as elevated liver enzymes, hepatitis, or hepatic veno-occlusive disease. Thiopurine-induced pancreatitis is another adverse effect of unknown origin, which occurs in less than 5% of patients treated with AZA or 6MP, mainly in the first month of treatment [47]. The most common reasons for thiopurine discontinuation are gastrointestinal disturbances such as nausea, vomiting and abdominal pain [48].

Much attention is paid to the adverse effects of combination therapy in IBD patients. Treatment with both IFX and thiopurines is known to generate specific adverse effects. Particular concerns stem from the risk of infection and cancer formation. Data on the safety of this form of treatment come mainly from a pooled post hoc analysis of drug registration trials, dedicated trials on combination therapy, and registries. It has been shown that patients treated with IFX in combination with immunosuppressants have a higher incidence of infections (120.07 (95% CI 110.66, 130.08)/100 patient-years vs. 92.47 (84.54, 100.94)/100 patient-years). Patients with CD treated with immunosuppressants had a higher incidence of malignancies compared to the absence of immunosuppression treatment (1.84 (0.22, 6.66)/100 patient-years vs. 0.00 (0.00, 0.00)/100 patient-years) [49].

Clinical registries are beneficial in assessing the safety of drugs. Firstly, they cover a larger group of patients compared to randomized trials. Secondly, they represent real clinical practice. The Crohn’s Therapy, Resource, Evaluation, and Assessment Tool (TREAT^™^) was designed to assess the safety of drugs in CD. This registry included 6273 patients. The median follow-up was six years. It showed that the use of immunosuppressants as monotherapy (OR 4.19; 95% CI 0.58–30.37; *p* = 0.16) or in combination with IFX (OR 3.33; 95% CI 0.46–24.06; *p* = 0.23) was associated with a numerically greater risk of malignancy than treatment with IFX alone (OR 1.96; 95% CI 0.23–17.02), although it was not statistically significant (*p* = 0.54) [50]. Similar results were obtained in other long-term studies of adalimumab (ADM) safety in CD [51]. The PYRAMID registry showed that ADM monotherapy vs.combination therapy showed a marked difference in the incidence of malignancies. In addition, there was a significant difference in the incidence of serious infections between ADM monotherapy and combination therapy (9.6 vs. 12.7%, *p* = 0.007) [52]. Limited data are available on the safety of combining immunosuppressants with vedolizumab. However, based on available investigations, no combination therapy was shown to lead to an increase in adverse effects [53]. Moreover, combined therapy and thiopurine monotherapy resulted in a significantly higher proportion of patients with severe COVID-19 compared to TNF-antagonist monotherapy (8.8% and 9.2% vs. 2.2%, respectively, *p* < 0.001). The comparative analysis of TNF-antagonist monotherapy, combination therapy (adjusted OR 4.01, 95% CI 1.65–9.78), and thiopurine monotherapy (adjusted OR 4.08, 95% CI 1.73–9.61) showed a significantly increased risk of severe COVID-19 [54].

In summary, the combination of AZA with anti-TNF antibodies increases the effectiveness of the therapy. In patients who start therapy with IFX, combination therapy is recommended for about a year. During this treatment, doses of immunosuppressants should be lower than in monotherapy. In patients who discontinue biological treatment, it seems advisable to continue treatment with AZA. However, the potential risk of adverse effects should be assessed in all cases.

### 4.5. Thiopurine Cytotoxicity and Pregnancy in IBD

The safety of thiopurines in pregnancy has long been a controversial topic. There has long been evidence that both AZA, 6MP, and TG and their metabolites pass through the placenta to the fetus [55]. At the same time, a significant and positive correlation between infant and maternal 6TGN level at delivery was demonstrated. The last data including 40 pregnant IBD patients on thiopurines revealed that at delivery, the median 6TGN level was lower in infants than mothers in a ratio of 0.4:1 (78.5 vs. 217 pmol/8 × 108 RBCs, *p* < 0.001) [56].

The current state of knowledge shows that conventional thiopurine exposure throughout conception and pregnancy is considered safe and is not associated with a higher risk of preterm birth or congenital disorders [57,58]. Recently, Mahadevan et al., basing their analysis on prospective multicenter studies among 1490 completed pregnancies, demonstrated that thiopurines, anti-TNF drugs, or combination therapy during pregnancy were not associated with increased adverse maternal or fetal outcomes at birth or in the first year of life. Moreover, the data obtained by those authors confirm the impact of higher disease activity on adverse effects (spontaneous abortion, hazard ratio 3.41, 95% CI 1.51–7.69; and preterm birth with increased infant infection, OR 1.73, 95% CI 1.19–2.51) [59]. Therefore, clinical remission in IBD patients for at least a couple of months before conception and during pregnancy is significant to reduce the risk of spontaneous abortion and premature birth, and to promote reaching a healthy weight [60]. 

Nevertheless, in patients with IBD who are planning a pregnancy, particular attention should be paid to the level of metabolites of thiopurine drugs in the red blood cells (RBCs) and to the use of the available pharmacogenetic tools, i.e., determination of *TPMT* and *NUDT15* gene alleles [61]. 

### 4.6. Solutions to Cytotoxicity and Resistance to Thiopurines

In IBD patients, resistance to thiopurines and potent therapy cytotoxicity can be overcome by using a split dose of AZA or mercaptopurine (e.g., 50 mg twice a day instead of 100 mg once daily) calculated using the conventional weight-based dosing approach (AZA 2–2.5 mg/kg, 6MP 1–1.5 mg/kg). This solution was first described in 2012. On the one hand, it reduces 6-MMP metabolites and on the other, it maintains 6TGN levels, serving as an effective strategy to preserve immunomodulator therapy in IBD patients who have a preference for 6-MMP metabolism [46]. 

Another strategy is a combination of AZA or 6MP with allopurinol, an inhibitor of the XDH enzyme that saturates or reduces the TPMT methylation capacity (Figure 1). Several studies demonstrated a significant reduction in 6-MMP and an increase in the 6TGN level and clinical remission and mucosal healing of therapy in nearly half of IBD patients intolerant to conventional thiopurine therapy. However, numerous opportunistic infections occurred [62,63,64]. At present, the effects of allopurinol on the thiopurine metabolic pathway itself are still unknown. There are some hypotheses that this drug may damage HPRT or play a role in the methylation of thiopurines [65,66].

**Figure 3:**
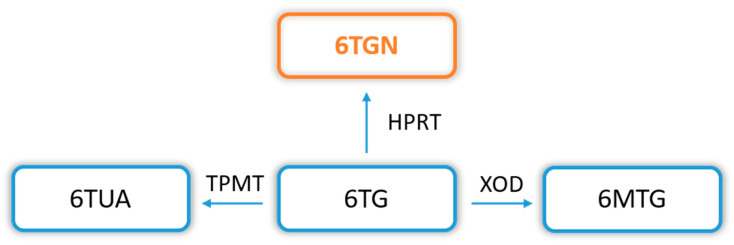
6-thioguanine (6TG) biotransformation pathway. Explanation of abbreviations: 6TG—6-thioguanine; TPMT—thiopurine S-methyltransferase; 6TUA—6-thiouric acid; HPRT—hypoxanthine phosphoribosyltransferase; 6TGN—6-thioguanine nucleotides; XOD—xanthine oxidase/dehydrogenase; 6MTG—6-methyl thioguanine.

In addition, a thiopurine alternative to common AZA and 6MP is also TG, which transformation pathway (to therapeutic TGN) is much shorter and has reduced cytotoxic potential. The conversion of TG to TGN requires only the participation of HGPRT, without ITPase, in contrast to the AZA and 6MP biotransformation (Figure 1 and Figure 3). 

Treatment with 6TG is approved by the European Medicines Agency and the US Food and Drug Administration as an alternative to conventional thiopurine therapy in treating acute nonlymphocytic leukemia and acute lymphoblastic leukemia [67]. However, this therapeutic approach is not quoted in the IBD international guidelines. 6TG use has been restricted in IBD due to its association with the subsequent development of nodular regenerative hyperplasia of the liver and portal hypertension. However, this complication was observed in patients receiving high doses of 6TG, of up to 100 mg/day [68]. A retrospective study on the long-term safety of 6TG therapy in 274 IBD patients, who previously failed therapy with conventional thiopurines, demonstrated a therapeutic effect in 51%, and good toleration as a maintenance treatment for IBD in about 70% of patients [69]. In contrast to AZA and 6MP, the dose of 6TG does not depend on the patient’s weight and it amounts to that administered in a daily portion (20 mg/day). The authors often indicated adverse events, but these were mainly mild or moderate. Therefore, 6TG in small doses is proposed as an effective therapy for IBD patients with a target threshold concentration of 6TGN ≥ 700 pmol/8 × 108 RBC [70]. 

Biemans et al. conducted a comparative analysis of 6TG vs. low-dose thiopurines combined with allopurinol in 182 IBD patients who failed conventional thiopurine therapy due to adverse effects [69]. They observed that 19% of patients discontinued treatment due to adverse effects. In other words, in 81% of patients, the solution with 6TG and low-dose thiopurines combined with allopurinol allowed them to avoid adverse events. These therapies can be considered as comparable before biological treatment [69]. Interestingly, it was recently found that gut microbiota can convert 6TG to therapeutically effective 6TGN, resulting in an additional means of local immunosuppression [71]. These findings were confirmed in animal experiments [67,72].

Summarizing the issue of improving the effectiveness of thiopurines therapy and finding a solution to their cytotoxicity, we should remember that in the case of confirmed decreased activity of the main enzyme TPMT and NUDT15, the guidelines of the Clinical Pharmacogenetics Implementation Consortium (CPIC) recommend reducing AZA, 6MP, or 6TG, or considering an alternative non-thiopurine immunosuppressant therapy [73]. A separate chapter of this review is devoted to assessing cytotoxicity and searching for predictive biomarkers.

## 5. Assessment of the Cytotoxicity of Thiopurine Drugs in IBD Patients

The balance between efficacy and cytotoxicity of the therapy should be achieved with appropriate dosing and monitoring. Thiopurines are dosed using a weight-based regimen. However, it is known that the dose of the drugs does not correlate with the level of metabolites [74]. Could monitoring thiopurine metabolites and enzyme activity enable personalized dosing in patients who entered treatment? What is the current knowledge of pharmacogenetic markers as predictors before treatment? These issues will now be discussed.

### 5.1. Thiopurine Metabolite Levels 

Measuring the metabolite level allows the efficacy and potential toxicity of thiopurine treatment to be evaluated. This is currently the most well-studied potential biomarker for predicting response to thiopurine treatment [75]. For this purpose, the high-performance liquid chromatography–ultraviolet spectroscopy (HPLC-UV/VIS) in human blood with values expressed in pmol/8 x 108 RBCs was adapted to measure the level of 6TGN and 6MMP [76]. The therapeutic range for use in clinical practice is 235–400 pmol/8 × 108 RBCs for 6TGN, and less than 5700 pmol/8 × 108 RBCs for 6MMP is considered a decreased risk of hepatotoxicity. This level was shown to correlate with efficacy and was associated with clinical response and remission in thiopurine monotherapy [12,77]. Unfortunately, the optimal use of thiopurine metabolite levels to achieve the maximal response in IBD patients is not a validated protocol. This varies between countries, and the optimal 6TGN cutoff when thiopurines are used in combination with anti-TNF agents has not been determined [2,78]. 

The other drawback to the widespread use of thiopurine metabolites in diagnostics is the limited availability of analytical testing. The therapeutic monitoring of 6TGNs and other metabolites is not practical for every clinic [79]. In everyday medical practice, parameters such as Crohn’s disease activity indices (CDAI) for patients with CD, the Truelove–Witts score for patients with UC, cell counts (white blood cells and absolute neutrophil count), and serum transaminases (alanine aminotransferase (ALT) and aspartate aminotransferase (AST)) are analyzed to estimate thiopurine treatment efficiency and adverse effects. Therefore, observations and comparative analyses of hematological parameters, such as the mean corpuscular volume (MCV), MCV at 9th week-baseline MCV (ΔMCV), and macrocytosis, are carried out to identify surrogate markers for monitoring the concentration of thiopurine metabolites during therapy. However, there are associations between, e.g., macrocytosis and an elevated MCV with 6TGN. Nevertheless, these are not strong biomarker correlations [80].

Returning to the topic of standard thiopurine metabolite measurement in IBD patients using HPLC-UV/VIS, it is necessary to present the third limitation of those methods, namely specificity and resolution. Here the level of 6TGN is indicated indirectly with the designation of 6TG. At the same time, the concentration of MTIMP is represented by 4-amino-5-(methylthio)carbonyl imidazole [81]. Standard HPLC is not able to distinguish between the mono-, di-, and triphosphates of 6TGN, ribosides, and deoxy analogs and presents them in sums. The modifications carried out by Vikingson et al. enabled the characterization of the metabolite patterns in more detail. Their results showed that 6TGN measurements using the routine method were consistently lower and, on average, 69% of calculated 6TGN values were measured by the specific method as a sum of 6TGTP (85% of total TGN), 6TGDP (14%), and 6TGMP (up to 2.4% of total TGN). The discrepancy probably stems from improved detection sensitivity of HPLC techniques and differences in the sample preparation procedure. 6TGMP, 6TGDP, and 6TGTP were identified in the modified method by fluorescence detection (excitation at 330 nm and emission at 410 nm) and 6MTIMP by UV detection at 289 nm. By assuming 100% hydrolysis, the 6MTIMP concentrations found using the modified method represent 19% (range 14–29) of the concentrations found with the routine procedure, as about 80% come from one or more additional metabolites [82]. 

The next step for increasing the quality and sensitivity of thiopurine active metabolites analysis in IBD patients was using liquid chromatography and detection by tandem mass spectrometry (LC-MS). Coulthard et al. developed a sensitive assay to quantify deoxythioguanosine (dTG) without derivatization in the DNA of nucleated blood cells. They assumed that incorporating dTG into the DNA may be a more important marker of the therapeutic response. The authors did not observe a correlation between dTG levels and drug dose and, due to the small number of patients, strong conclusions could not be drawn. However, dTG was not detected in the DNA of patients not treated with AZA. Therefore, further studies are necessary to explain the suitability of dTG determination in monitoring thiopurine treatment and its adverse effects [31].

In research on in vitro cell models treated with AZA and 6MP, a methodology enabling the identification and quantification of the concentration of the metabolites with cytotoxic activity was developed. For this purpose, human hepatocytes and intestinal epithelial cells were used [83,84]. The data that were obtained showed that innovative mass spectrometry assay enables the simultaneous evaluation of 11 metabolites with different mono-, di-, and triphosphate thionucleotides, i.a., 6TIMP, 6TGMP, 6TGDP, 6TGTP, 6MTIMP, 6MTIDP, and 6MTITP. Pelin et al. reported that methylthioinosine monophosphate (MTIMP) concentrations were associated with lower hepatocyte survival, and the ratio between MTIMP and 6TGMP metabolites directly correlated with cell survival [85]. Recently, Genova et al. described a dependence of the in vitro cytotoxicity on the 6TGMP, 6TGDP, 6TGTP, and methylthioinosine triphosphate (MTITP) concentrations after the four-hour incubation before the addition of thiopurines [86]. The results of both these publications provide in vitro models for studying thiopurine liver and intestinal cytotoxicity, which is especially valuable because of the need for further research on thiopurine drug metabolites.

### 5.2. Enzyme Activity 

Over many years, enzyme activity has been assessed at the phenotype and genotype level to develop methods useful in pre-drug practice for predicting patient responses. The first study measuring TPMT activity in humans was reported in 1980 by Weinshilboum and Sladek. By examining a cohort of 298 randomly selected Caucasians, the authors demonstrated trimodal erythrocyte TPMT enzyme activity distribution: 11.1% with intermediate activity, 89.6% with high activity, and 0.3% with no activity. Those results were associated with an autosomal codominant inheritance of a pair of alleles for low and high TPMT activity and were a breakthrough in pharmacogenetics [87].

Pre-treatment determination of TPMT genotype and phenotype may be helpful for predicting thiopurine toxicity. However, evidence of their value is still unclear. For example, patients with TPMT activity of less than 30.5 EU/mL were more likely to have a clinical response to thiopurines than those with higher activity [85]. Other studies did not confirm this dependence [88]. The majority of adult patients with myelotoxicity had a normal TPMT genotype. There is also inaccuracy between genotype and phenotype TPMT activity [89]. Genotyping sensitivity significantly ranged in patients with intermediate and low enzymatic activity [90]. On the contrary, genetic polymorphism has been reported to play a pivotal role in thiopurine adverse drug reactions. 

In addition, the presence of a large number of enzymes of the thiopurine biotransformation pathway and the variability of their activity provides a rationale for measuring the level of metabolites in the blood as an effect of drug metabolism and distribution to determine the optimal dose of drugs for each IBD patient and to avoid toxicity.

### 5.3. Genes Implicated in Thiopurine-Induced Toxicity in IBD Patients 

A great deal of data exists on research into the pharmacogenetics of thiopurine drugs. The challenge of personalized medicine is to predict the effects of therapy in advance by analyzing genetic variants and individually adjusting the medication and dose. In the case of thiopurine drugs, the complex biotransformation pathway of AZA and 6MP is an impediment. On the other hand, the associations between *TPMT* gene alleles and the metabolic rate are powerful and recognized by the FDA as one of the first major pharmacogenetic biomarkers.

**Figure 4:**
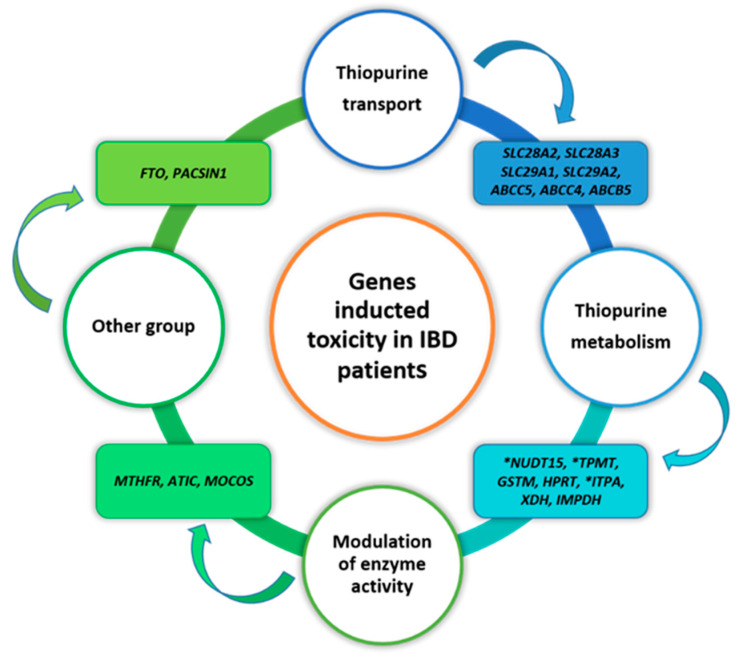
Potential genes inducing cytotoxicity in IBD patients. Explanation of abbreviations: *—widely characterized impact; *SLC28A2*—solute carrier family 28 member 2; *SLC28A3*—solute carrier family 28 member 3; *SLC29A1*—solute carrier family 29 member 1; *SLC29A2* solute carrier family 29 member 2; *ABCC5*—ATP binding cassette subfamily C 28 member 5; *ABCC4*—ATP binding cassette subfamily C member 4; *ABCB*5—ATP binding cassette subfamily B member 5; *NUDT15*—nudix hydrolase 15; *TPMT*—thiopurine S-methyltransferase; *GSTM*—glutathione S-Transferase Mu; *HPRT*—hypoxanthine phosphoribosyltransferase; *ITPA*—inosine triphosphatase; *XDH*—xanthine dehydrogenase; *IMPDHA*—inosine-5′-monophosphate dehydrogenase; *MTHFR*—methylenetetrahydrofolate reductase; *ATIC*—5-aminoimidazole-4-carboxamide ribonucleotide formyltransferase; *FTO*—alpha-ketoglutarate dependent dioxygenase; *MOCOS*—molybdenum cofactor sulfurase; *PACSIN1*—protein kinase C and casein kinase substrate in neurons 1.

The genes implicated in thiopurines-induced toxicity in IBD patients can be classified into four different groups, as presented in Figure 4.

To date, the best-characterized genes encoding thiopurine metabolic pathways are *TMPT*, *ITPA*, *HPRT*, *XDH*, *GSTM1*, *XDH*, *GMPS*, and *NUDT15* [91]. However, the effects of *TPMT* and *NUDT15* genes on the impact of treatment with thiopurines and its adverse effects have been documented. Hence recommendations have been developed for adjusting the starting doses of AZA, 6MP, and 6TG based on *TPMT* and *NUDT15* genotypes, and testing of genetic polymorphisms in IBD patients has been implemented in clinical practice [73]. Currently, the Swedish Committee on *TPMT* Nomenclature Gene (https://liu.se/en/research/tpmt-nomenclature-committee, access date: 21 March 2022) presents 44 known variants of S-methyltransferase alleles. The polymorphisms described in the research showed the influence on tolerance, and the effectiveness and risk, related to the toxicity of thiopurine therapy [91]. The cytotoxic effect of thiopurines, in most cases in Caucasians, depends on the presence of the TPMT *3A allele (c.460G > A, p.Ala154Thr; c.719A > G, p.Tyr240Cys). At the same time, in Latinos and Asians, it is the variant c.719A > G [92,93]. Clinical trials described in 2018 involving 219 IBD patients assumed a comparison of the main predictors. The results showed a difference between Caucasians and Asians [94,95]. 

The *NUDT15* gene, c.415C > T (rs116855232) polymorphism, was proved to be a critical genetic factor of AZA-induced leukopenia in Chinese patients. The C/T genotype was detected in 44 patients, 16 received AZA, and 50% developed severe leukopenia. Homozygotes C/C were associated with a 17.2% risk of developing inflammation related to the depletion of leukocytes [96]. NUDT15 is responsible for the hydrolysis of 6TG triphosphate to monophosphate and its final deactivation. Polymorphism p.R139C and variants T/T and C/T are examples of reduced activity and of the incorporation of toxic metabolites into DNA [86]. The results confirmed the findings of previous studies that the *TPMT* gene determines leukopenia and myelosuppression in European patients [97]. NGS sequencing and clinical tests identified the *TPMT* gene polymorphism, which led to liver damage in 10% of patients with a deficient functional allele and 21% of heterozygotes undergoing treatment for inflammatory diseases [98,99]. Genetic variability leads to a decreased activity of the TPMT enzyme and thus influences the overproduction of the critical metabolite 6TGN. As a result, the bone marrow is disturbed and the synthesis of mature leukocytes is limited [100]. 

Recent studies on a group of patients with IBD and other autoimmune diseases showed the importance of implementing a deeper analysis of genotyping patients [29,99]. A group of 107 European patients with the following missense variants in the *NUDT15* gene were considered significant: p.Gly17_Val18del, p.Val18_Val19insGlyVal, p.Arg139Cys, c.3G > C, c.217delA [101]. The newly discovered c.3G > C variant led to the loss of the start codon, while the c.217delA affected the reading frameshift. Of the 107 patients previously sequenced for a non-functional *TPMT* allele, 43% showed signs of anemia and leukopenia. The presence of a polymorphism for the *NUDT15* gene was demonstrated for 13% of the subjects, and in 6%, two variants of genes and severe hepatotoxicity were observed. The presented studies confirmed the influence of the *NUDT15* gene on the pharmacokinetics of thiopurine drugs before implementing thiopurine therapy in IBD patients [99]. The studies also set new goals for the future so that potential pharmacokinetic differences are tested for different ethnicities and even for a specific nationality. It is also worth noting the correlation between the simultaneous occurrence of both *TPMT* and *NUDT15* gene polymorphisms and severe cytotoxicity [102]. 

A 2020 study by Choi et al. was carried out in a group of 131 patients with IBD and included sequencing and statistical analysis of an additional 34 genes other than the main determinants: *NUDT15* and *TPMT*. Two polymorphisms of the *ATIC* gene demonstrated statistical significance (*p* < 0.05), the enzyme of which acts as a catalyst in the final stages of purine biosynthesis. Polymorphism rs3821353 was responsible for the level of 6TGN in cells, while variant rs16853834 and the rs11706052 *IMPDH2* gene were related to the ratio of the metabolite 6TGN and the dose of thiopurine administered. The level of 6MMPN metabolite and the maintenance doses of the drug were correlated with the activity of *ITPA* rs6139036 [103]. The last analysis showed the same result for the rs8362 polymorphism of the *ITPA* gene [104]. In the children with IBD and the group with other autoimmune disorders, the results showed a decrease in ITPA activity, potentially contributing to the accumulation of 6MMP [105,106]. 

In Asian population, the polymorphism c.400G > A of the alpha-ketoglutarate-dependent dioxygenase gene (*FTO*) was associated with a 65% decrease in its enzymatic activity and an increased likelihood of leukopenia [107]. The genetic variant rs16952570 may regulate the expression of the *FTO* gene. Higher white blood cells and neutrophils were observed in patients with C/C FTO genotypes compared to T/T homozygous one month after starting thiopurine treatment [108]. The polymorphism rs2647087 of the *HLA* gene is associated with pancreatitis during thiopurine therapy. In the non-functional C/C homozygote, the risk of inflammation was 14.6%, A/C heterozygote 4.3%, and wild-type A/A 0.5% [109]. The first clinical studies on the influence of the *MOCOS* gene on the thiopurine metabolic pathway showed its essential role in the enzymatic regulation of AOX1 and XDH. However, further analyses are still needed to fully elucidate the molecular mechanism of the *MOCOS* (rs594445) gene on cytotoxicity. The same study in a group of pediatric IBD patients indicated that the *GMPS* gene determined tolerance (*p* = 0.02) to the drug used. The induced cytotoxicity was caused by attaching a phosphate residue to 6-TIMP and its conversion into 6TGN [90]. In IBD, little is known about the impact of transporters. The polymorphism rs8180093 of the *ABCC5* gene, determining drug resistance to 6MP, significantly influenced leukopenia development in patients with IBD [103]. *SLC29* and *SLC28* genes are responsible for the uptake of thiopurine drugs and the transport and accumulation of cytotoxic substances. However, this effect has been described in acute lymphoblastic leukemia patients treated with thiopurines. Further research is needed to determine the exact correlation of their polymorphisms with the cytotoxic effect of thiopurines in IBD patients [104].

To summarize, the list of candidate genes implicated in thiopurine-induced toxicity in IBD patients seems long but remains imprecise. Many relationships found in genome-wide research require verification and functional studies. Nevertheless, pharmacogenetics is essential in predicting thiopurines’ adverse effects in IBD patients before therapy induction.

## 6. Future Perspective in Cytotoxicity Research of Thiopurine Drugs in IBD

Taking into account the constant need to use thiopurines in the treatment of IBD and the challenge of personalized medicine, the continuation of extensive pharmacogenetic research and metabolite monitoring seems natural and obvious [110,111]. On the other hand, as the human body contains more microbes than human cells, the microbiome (the ‘second human genome’) is of great interest to scientists. Thus, a wave of research on the interaction of gut microbiota and the effects of thiopurine treatment should be expected for IBD patients. Effenberger and coworkers support the validity of such a hypothesis, describing an in silico metabolic prediction analysis by including azathioprine or anti-TNF antibody-treated IBD groups. They assessed the effect of gut microbiota function on remission status and found that the predicted butyrate synthesis was significantly enriched in patients achieving clinical remission [112]. These findings suggest a functional link between microbiota and the efficiency of immunosuppressive therapy in IBD. The question remains as to whether the composition of the human microbiota may also influence the phenomenon of the cytotoxicity of thiopurines. 

## 7. Conclusions

The safety of using purine medications in the treatment of IBD patients has been the subject of intense debate for many years. The current knowledge supports an informed choice of treatment and the appreciation of the benefits of thiopurines in pregnant patients. This position stems from the fact that safety concerns with thiopurines are real but also relatively rare. Moreover, predictive pharmacogenetic screening is more efficient due to extending the *TPMT* gene analysis to *NUDT15*, and monitored thiopurine benefits outweigh the risks in the majority of appropriately selected patients, including pregnant ones. Further optimization of thiopurine dosing via measurement of their metabolites should be performed routinely and is superior to weight-based dosing. Intestinal microbiota may also prove to be an interesting subject of research in the context of the effectiveness of thiopurine therapy in patients with IBD.

## Data Availability

Not applicable.
